# Uncovering adulteration and quality variations in commercial lavender essential oils from the Egyptian market using GC–MS and chemometrics

**DOI:** 10.1038/s41598-026-45972-6

**Published:** 2026-04-18

**Authors:** Mostafa B. Abouelela, Eman M. El-Taher, Enas M. Shawky, Mostafa H. Baky

**Affiliations:** Pharmacognosy Department, Faculty of Pharmacy, Egyptian Russian University, Badr City, Cairo, 11829 Egypt

**Keywords:** Lamiaceae, Lavender, Essential oils, GC–MS, Chemometrics, Adulteration, Authentication, Quality control, Biochemistry, Chemistry, Plant sciences

## Abstract

Lavender (*Lavandula* L. spp., Lamiaceae) essential oil is widely valued for its therapeutic effects and aromatic properties. Owing to the growing global demand for this essential oil, the implementation of standardized quality assessment protocol is essential to ensure its quality. This study aimed to perform a comparative metabolite profiling of four commercially available lavender oils in the Egyptian market using Gas chromatography-mass spectrometry (GC–MS) analysis combined with chemometric analysis. A total of 54 volatile metabolites were identified belonging to 8 chemical classes including alcohols, aldehyde/ketone, esters, fatty acids/esters, oxides, aliphatic, monoterpene, and sesquiterpene hydrocarbons. Alcohols and esters represented the abundant classes accounting for 24.7–42.6% and 11.4–46.1%, respectively, of the total volatile composition. Linalool and linalyl acetate, the principal quality markers of lavender oil were comparatively higher in L-Sha (33.61, and 24.72%) and L-Rag (46.09, and 32.65%), indicating their higher quality among the examined samples. In contrast, certain samples exhibited unusually high levels of non-characteristic fatty acid esters and synthetic glycols, suggesting possible dilution or adulteration with carrier oils or formulation additives. Multivariate analysis further demonstrated clear compositional discrimination among samples, confirming substantial variability in the quality of commercial lavender oils available in the Egyptian market. These findings highlight the importance of GC–MS profiling combined with chemometric analysis as a robust tool for authenticity verification, quality control, and detection of potential adulteration in commercial essential oil products.

**Supplementary Information:**

The online version contains supplementary material available at 10.1038/s41598-026-45972-6.

## Introduction

The demand for natural products, particularly essential oils, has increased markedly in recent years due to their myriad applications in the medical, pharmaceutical, cosmetic, and industrial sectors^1^. Essential oils provide valuable therapeutic benefits owing to their antimicrobial, anti-inflammatory, and antioxidant properties^2^. In 2023, the global market of essential oil was valued at 23.74 USD billion and expected to grow at a compound annual growth rate of 7.6% between 2024 and 2030^3^. In Egypt, the essential oils exports reached USD 51.1 million in 2023, and forecasted to grow from 10.8% in 2025 to 15.8% by 2029^4,5^. Among the diverse essential oils, Lavenders (*Lavandula* spp.) represents a prominent example in which chemical variability can substantially influence both therapeutic efficacy and commercial value^6^. The global lavender essential oil market is expanding steadily, valued at USD 138.2 million in 2024 and projected to reach USD 267.2 million by 2034^7^.

Lavenders (*Lavandula* spp.) belongs to Labiatae (Lamiaceae) family, one of the largest and most economically important plant families, comprising approximately 236 genera and 7000 species^8^. *Lavandula* is a particularly important genus, featuring over 40 species along with numerous subspecies^6^. It has been utilized for centuries, either in dried form or as essential oil, for various therapeutic and cosmetic applications. Typically, lavender essential oil is extracted through steam distillation from both the flowers and leaves^9^. However, the chemical composition varies significantly depending on factors such as botanical origin, geographical location, cultivation conditions, and extraction method^10^. Lavender originated in the low mountainous regions of the Mediterranean, but it is now cultivated worldwide for essential oil production^6^. Lavender essential oil has been recognized for its antibacterial, antifungal, carminative, sedative, and antidepressant effects, and is considered beneficial for treating burns and insect bites^11^. Lavender essential oil is primarily characterized by the presence of linalool and linalyl acetate as its major constituents; however, its chemical composition may vary considerably due to genetic variability among oil-producing plants, environmental conditions, cultivation practices, and extraction methods. Furthermore, the phytochemical profile differs among species and hybrids of *Lavandula*, contributing to additional compositional diversity.

Commercial lavender oil is susceptible to adulteration with lower-cost essential oils or synthetic compounds^12^, which complicates authenticity assessment and quality control^13^. Common adulteration practices include dilution with inexpensive carrier oils^14^, addition of synthetic aroma compounds such as synthetic linalool or linalyl acetate, and substitution with oils from closely related species such as *Lavandula intermedia* (lavandin)^15^. These practices can significantly alter the natural chemical composition of authentic *Lavandula angustifolia* oil, affecting its therapeutic efficacy and market value.

The cultivation of lavender for commercial and industrial purposes has expanded rapidly in recent years because of heightened global demand^16^. Notably, the market price of commercially available lavender oil varies considerably, and such variability should ideally reflect differences in chemical quality and compositional integrity^17^. However, despite their popularity, ensuring the quality, authenticity, and safety of these oils remains a significant challenge^2^.

Despite the economic importance of essential oils in Egypt and the existence of national standards regulating their quality, including the Egyptian Organization for Standardization specification for lavender oil (EOS 4173/2008) challenges remain regarding the consistent evaluation of product quality and authenticity in commercially available preparations^18^. For lavender oil, variability in production practices, botanical sources, and commercial formulations may still lead to considerable differences in the chemical composition of marketed products^19,20^. Therefore, reliable analytical approaches are required to assess the authenticity and compositional quality of essential oils available in the marketplace. With the increasing consumer demand for well-characterized herbal products, advanced analytical techniques have become essential for evaluating their chemical profiles and ensuring quality consistency^21^. In this context, metabolomics-based approaches, particularly gas chromatography-mass spectrometry (GC–MS) combined with multivariate chemometric analysis, have emerged as powerful tools for comprehensive profiling of complex volatile metabolite matrices and for the comparative assessment of samples obtained from different sources or processing methods^22^. Accordingly, the main objective of the present study was to investigate the volatile composition and quality variation of four commercially available lavender essential oils (L-Imt, L-Nef, L-Sha, and L-Rag) widely consumed in the Egyptian market using GC–MS profiling coupled with chemometric analysis.

## Results and discussion

**Fig. 1:**
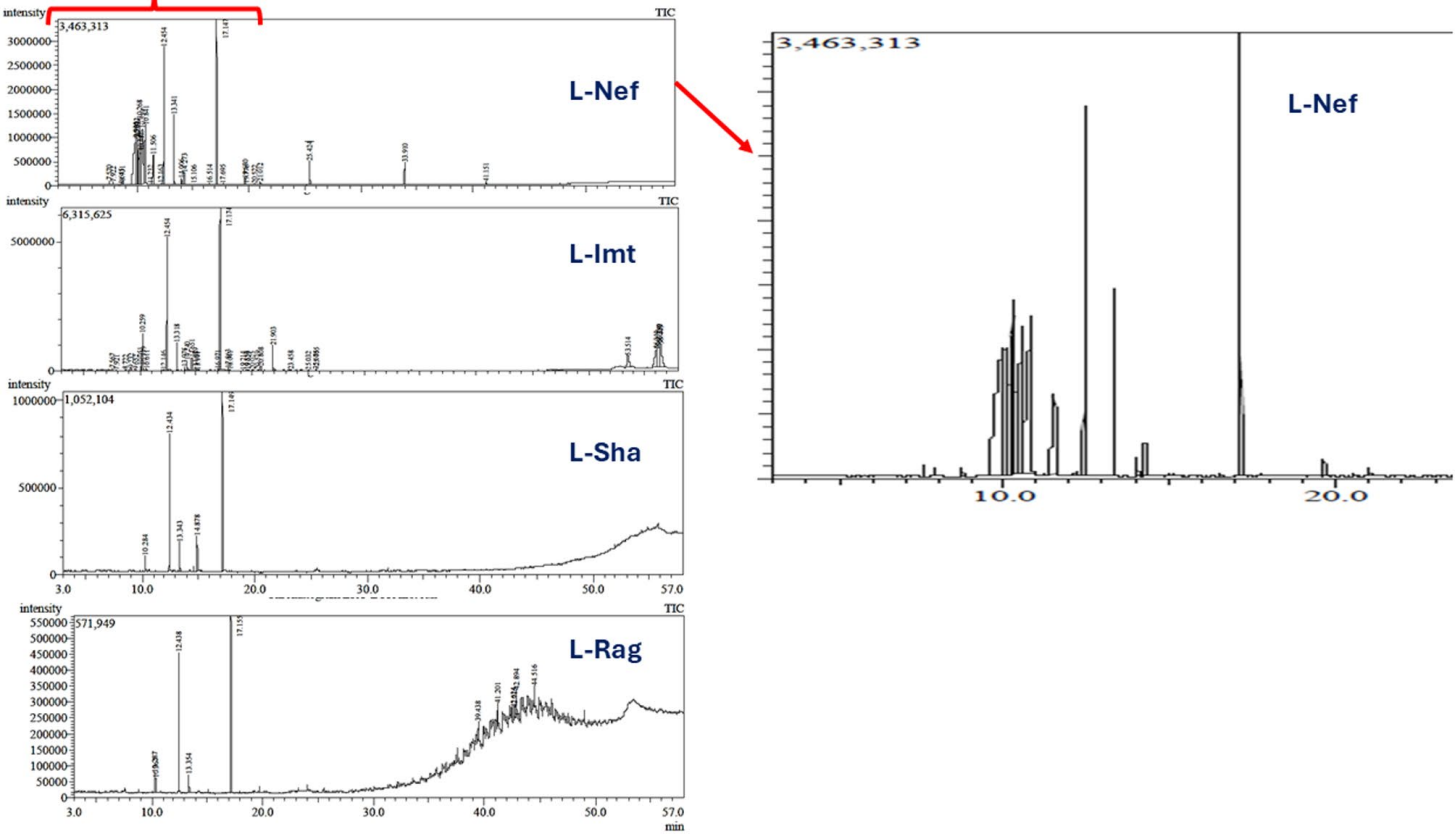
GC–MS chromatograms illustrating the volatiles identified across the four commercial products.

**Fig. 2:**
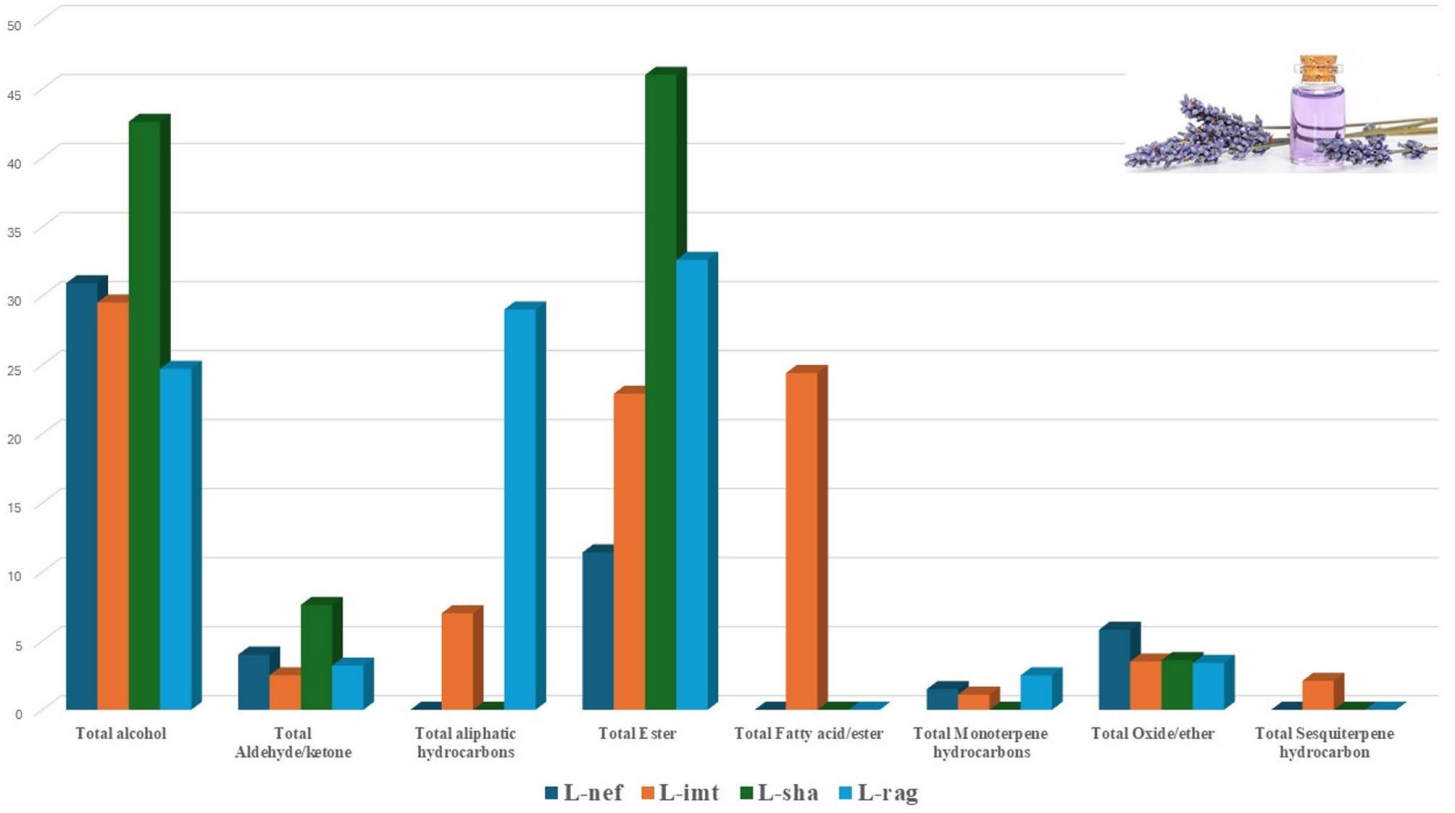
Distribution patterns of volatile compound classes detected in the four products.

GC–MS analysis revealed annotation of 54 volatile metabolites belonging to 8 chemical classes, viz*.*, alcohols, aldehydes, aliphatic hydrocarbons, esters, fatty acids, monoterpene hydrocarbons, oxides, ethers, sesquiterpene hydrocarbons (Table 1). Representative chromatograms of the four products are illustrated in Figs. 1 and S1, and the relative distribution of the metabolite classes is summarized in Fig. 2. Chromatogram peak intensities reflect detector response and do not directly correspond to the normalized relative percentages reported in Table 1, which were calculated from integrated peak areas. All samples were analyzed under identical conditions, with three replicates each, to ensure reproducibility.

**Table 1:** Chemical composition in four commercial lavender oil products analyzed via GC–MS (n = 3) and calculated as relative area percentage (%) of volatile metabolites.

No	R_t_ (Min)	RI	Compound name**	L-Nef	± Sd*	L-Imt	± Sd*	L-sha	± Sd*	L-Rag	± Sd*
1	7.57	948	α-Pinene	0.20	0.02	0.13	0.03	–	–	–	–
2	7.925	943	Camphene	0.10	0.01	0.03	0.02	–	–	–	–
3	8.645	1031	Limonene oxide	0.02	0.04	–	–	–	–	–	–
6	8.733	897	Sabinen	0.14	0.03	–	–	–	–	–	–
4	9.232	943	β-pinene	–	–	0.16	0.02	–	–	–	–
5	9.649	984	Hexyl acetate	–	–	0.02	0.03	–	–	–	–
7	9.935	843	2,4-Pentanediol	**16.21**	2.60	–	–	–	–	–	–
8	10.051	1042	p-Cymene	0.20	0.02	0.38	0.05	–	–	–	–
9	10.267	1059	Cineole	5.79	0.63	2.91	0.62	3.65	0.05	3.44	0.21
11	10.346	1143	α-Terpineol	2.72	2.36	–	–	–	–	–	–
10	10.347	1018	Sylvestrene	0.81	1.40	0.40	0.06	–	–	2.45	0.20
12	10.609	976	Ocimene	–	–	0.05	0.08	–	–	–	–
13	11.232	998	γ-Terpinene	0.01	0.02	–	–	–	–	–	–
14	12.165	1155	a-Campholenal	0.07	0.01	0.05	0.05	–	–	–	–
15	12.454	1082	Linalool	**10.91**	0.41	**18.59**	4.62	**33.61**	0.26	**24.72**	1.88
16	13.341	1121	Camphor	3.96	0.25	2.44	0.31	7.63	0.10	3.24	0.29
17	14.006	1138	Isoborneol	0.36	0.03	0.35	0.03	–	–	–	–
18	14.273	1138	Borneol	0.69	0.05	0.95	0.06	–	–	–	–
19	14.651	1137	Terpinen-4-ol	–	–	1.19	0.10	–	–	–	–
20	14.876	1195	Gardenol	–	–	–	–	9.02	0.04	–	–
21	14.989	1082	trans-Ocimenol	–	–	0.24	0.21	–	–	–	–
22	15.051	1220	Citronellol epoxide	–	–	0.01	0.02	–	–	–	–
23	15.106	1172	Estragole	0.03	0.06	–	–	–	–	–	–
24	15.191	1118	Hexyl isobutyrate	–	–	0.02	0.04	–	–	–	–
25	16.514	1335	Isopulegol acetate	0.06	0.01	–	–	–	–	–	–
26	16.971	1228	Nerol	–	–	0.19	0.02	–	–	–	–
27	17.147	1272	Linalyl acetate	**10.97**	0.45	**22.42**	4.45	**46.09**	0.30	**32.65**	2.37
28	17.696	1092	Dihydrolinalool	0.05	0.01	–	–	–	–	–	–
29	17.863	1277	Bornyl acetate	–	–	0.21	0.02	–	–	–	–
30	18.041	1404	Lavandulyl isobutyrate	–	–	0.09	0.01	–	–	–	–
31	19.214	1126	Hexenyl isobutanoate	–	–	0.02	0.02	–	–	–	–
32	19.558	1346	Epoxy-α-terpenyl acetate	–	–	0.07	0.06	–	–	–	–
33	19.68	1333	α-Terpineol acetate	0.35	0.03	–	–	–	–	–	–
34	19.776	948	2-Carene	0.04	0.03	–	–	–	–	–	–
35	20.025	1270	Lavandulyl acetate	0.02	0.03	0.10	0.08	–	–	–	–
36	20.808	1221	Copaene	–	–	0.39	0.03	–	–	–	–
37	21.903	1494	Caryophyllene	–	–	1.75	1.51	–	–	–	–
38	23.458	1128	α-Limonene diepoxide	–	–	0.05	0.05	–	–	–	–
39	25.032	1281	Isoaromadendrene epoxide	–	–	0.01	0.02	–	–	–	–
40	25.755	1507	Caryophyllene oxide	–	–	0.49	0.12	–	–	–	–
41	37.445	2804	Octacosane	–	–	–	–	–	–	1.06	1.84
42	37.603	3500	Pentatriacontane	–	–	3.88	6.71	–	–	2.11	1.83
43	39.438	2109	Heneicosane	–	–	0.00	0.00	–	–	3.71	0.18
44	41.201	3600	Hexatriacontane	–	–	0.11	0.18	–	–	4.37	3.80
45	42.624	3997	Tetracontane	–	–	–	–	–	–	2.43	0.31
46	42.7	2009	Eicosane	–	–	–	–	–	–	3.75	0.07
47	42.894	3401	Tetratriacontane	–	–	–	–	–	–	6.25	0.37
48	44.516	4395	Tetratetracontane	–	–	–	–	–	–	5.65	0.68
49	49.65	3997	Tetracontane	–	–	0.65	1.13	–	–	–	–
50	50.025	2228	Isopropyl linolate	–	–	2.40	4.16	–	–	–	–
51	50.525	3508	17-Pentatriacontene	–	–	2.46	4.26	–	–	–	–
52	50.74	3942	1-Heptatriacontanol	–	–	7.95	13.77	–	–	–	–
53	51.62	2963	Phytyl decanoate	–	–	1.25	2.16	–	–	–	–
54	53.514	2185	Oleyl acetate	–	–	12.08	20.92	–	–	–	–
Total alcohol				30.93	5.46	29.46	18.81	42.63	0.29	24.72	1.88
Total Aldehyde/ketone				4.03	0.26	2.49	0.36	7.63	0.10	3.24	0.29
Total aliphatic hydrocarbons				–	–	7.09	12.29	–	–	29.32	9.07
Total Ester				11.40	0.53	22.96	4.72	46.09	0.30	32.65	2.37
Total Fatty acid/ester				–	–	24.39	25.00	–	–	–	–
Total Monoterpene hydrocarbons				1.50	1.52	1.14	0.26	–	–	2.45	0.20
Total Oxide/ether				5.85	0.73	3.49	0.83	3.65	0.05	3.44	0.21
Total Sesquiterpene hydrocarbon				–	–	2.14	1.55	–	–	–	–
Total identified (%)				53.71		84.50		100.00		95.82	
Total Non-identified (%)				46.29		15.50		0.00		4.18	

Bold numbers indicates the most abundant compounds.

*Sd: standard of deviation, results calculated as mean ± Sd.

**Compounds are listed in order of their elution and their percentages were obtained by peak-area normalization.

### Alcohols

Alcohols were identified as the most abundant class in all samples accounting for 24.7%, 29.4%, 30.9%, and 42.6%, in L-Rag, L-Imt, L-Nef, and L-sha, respectively. Linalool (Peaks 15), a characteristic monoterpene of lavender oil^23^, previously reported in various lavender species^23^, was detected in all samples within a concentration range of 10.9–33.6%. Higher linalool levels were detected in both L-Rag (24.7%) and L-Sha (33.6%) consistent with profiles reported authentic *Lavendula angustifolia* oils. Linalool is one of the predominant floral scents present in lavender oil with a myriad of biological activities including sedative, anxiolytic, analgesic, antimicrobial, anticonvulsant, and anti-proliferative effects^24^. Owing to its antimicrobial activity, it could be used in cosmetic preparations as a preservatives^25^. Previous studies on lavender oils cultivated in Egypt have also reported variability in the relative abundance of the main constituents in the different species such as linalool (39.5%), lianlyl acetate (26.7%), linalyl acetate (46.41%)^26^. Moreover, GC–MS analysis of the chemical composition of essential oils of *L. angustifolia* and *L. hybrida* cultivated in Egypt revealed the abundance of monoterpenes, alcohols and esters^27^.

Several minor alcohols were differentially distributed among samples. 1-Heptatriacotanol (Peak 52), a long chain fatty alcohol, shown to possess several biological activities antioxidant anti-inflammatory, anticancer, anti-hypercholesterolemic, and antimicrobial effect^28^ it was detected only L-Imt with concentration 7.95%. Likewise, gardenol (peak 20), was detected only on L-sha. It is a flavor ingredient approved in many countries as a sweet and honey flavoring agent previously detected in clove bud oil^29^. Three additional alcohols including 2,4-pentanediol and α-terpineol, dihydrolinalool (Peaks 7, 11, and 28) were detected only at L-Nef with concentrations of 16.2%, 2.7% and 0.05% respectively.

The presence of 2,4-pentanediol (16.2%), , and α-terpineol (2.7%), a monoterpene alcohol, reported from lavender^23^ was unique for L-Imt. α-Terpineol previously reported with several biological activities such as antibacterial^30^, anti-inflammatory^31^ Analgesic^32^, and gastroprotective effect^33^. Dihydrolinalool, a hydrogenated derivative of linalool, is with floral, fruity odor^34^.

Trace amounts of borneol and isoborneol presents only in both L-Nef, and L-Imt, reported previously in *L. angustifolia*^23,35^. Borneol known for its biological activities such as antipyretic, antispasmodic, anthelmintic, and diaphoretic^35^. It was detected at range levels of 0.69%, and 0.95% in L-Nef, and L-Imt respectively.

Isoborneol, is characterized by spicy and fruity flavor, commonly used as a food additive and was detected with 0.35%, and 0.36% in L-Imt and L-Nef respectively. Terpinen-4-ol (peaks 19), trans-ocimenol (peaks 21), and nerol (peak 26) are detected at L-Imt with concentrations 1.19%, 0.24%, and 0.19%, respectively. Terpinen-4-ol, reported previously from *lavendulan angustifolia*^36^, exhibited several biological activity such as antimicrobial, antiviral, anticancer, antioxidant, anti-inflammatory, cardioprotective and anti‐hypertensive effect^37^. Moreover, trans-ocimenol, previously reported with sweet fruity odor and a higher degree of sweet citrus and rose-like fragrance has been associated with antifungal activity^38^. Also, Nerol reported from lavender^36^, possesses antioxidant, antifungal, antispasmodic, anthelmintic effects^39^, and anticancer activities^40^.

Variations in the relative abundance of certain minor alcohols may partly reflect natural phytochemical variability arising from genetic factors, geographical origin, climatic conditions, and agronomic practices, which are known to influence the biosynthesis of monoterpenes in lavender plants. For example, the relative ratios between key monoterpenes such as linalool, camphor, and terpinen-4-ol may vary among *Lavandula* species reported in the literature^41^. However, the occurrence of certain compounds in unusually high proportions or restricted to specific samples may also indicate non-natural influences related to formulation or adulteration practices. In particular, the presence of 2,4-pentanediol at a high relative abundance (16.2%) in L-Nef is atypical for authentic lavender essential oil composition. Glycols such as 2,4-pentanediol are not natural constituents of lavender oil and are more commonly associated with synthetic additives or formulation solvents used in fragrance preparations^42^. Similarly, the detection of several alcohols exclusively in individual samples, such as dihydrolinalool or α-terpineol, may reflect post-processing modifications, oxidative transformations, or the incorporation of fragrance additives, rather than natural plant-derived variability alone^43^. It could be deduced that, while the dominance of linalool supports the botanical identity of lavender in all analyzed products, the unusual presence or elevated levels of certain non-typical alcohols suggests that both natural compositional variation and potential commercial modification contribute to the observed chemical diversity among the examined oils.

### Esters

**Fig. 3:**
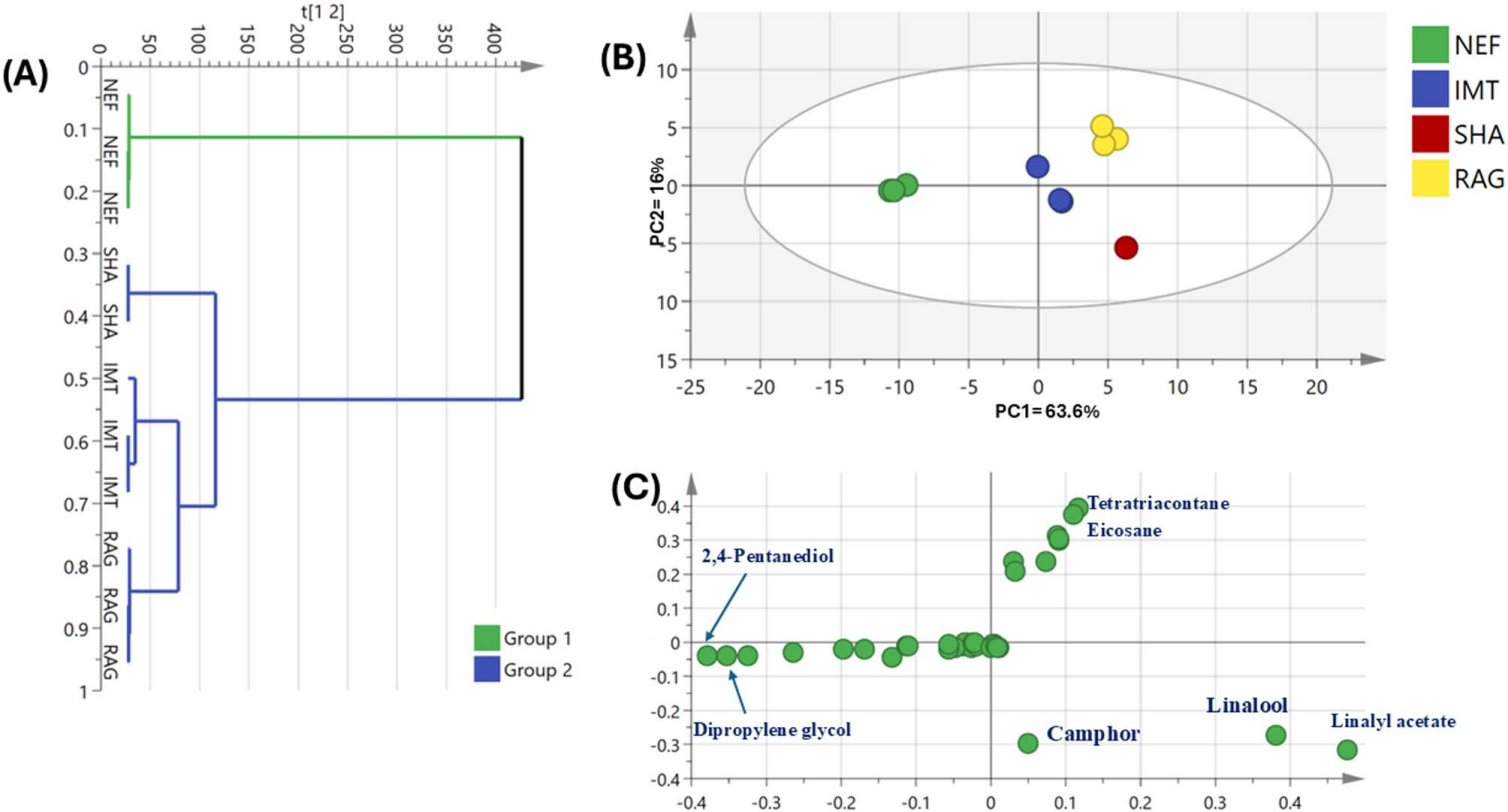
Unsupervised multivariate analyses of volatile compounds in lavender oil samples detected by GC–MS (n = 3). (**A**) HCA dendrogram. (**B**) PCA score plot of PC1 versus PC2 from GC–MS data. (**C**) Corresponding loading plot for PC1 and PC2 with peak assignments.

Esters represented the second most predominant chemical class contributing 11.4, 22.9, 32.6, and 46.1% in L-Nef, L-Imt, L-Rag, and L-Sha respectively (Fig. 3). Linalyl acetate (LA, Peak 27), the principal ester of lavender oil, is recognized for their numerous biological activities, including anti-inflammatory, anti-hypertensive, anti-hyperglycemic, antioxidant and neuroprotective effects^44^. Additionally, LA may prevent the onset of mild cognitive impairment^45^, and exerts substantial protective effects through its anti-hypertensive properties and its influence on eNOS expression^46^. The relative abundance of LA is a critical determinant of lavender oil quality as it significantly contributes to the characteristic sweet, floral aroma. The elevated concentration of LA was observed in L-Sha and L-Rag at *ca*. 46.09% and 32.65%, respectively, closer conformity to recognized lavender oil compositional profiles. Traces amount of bornyl acetate, and lavandulyl acetate, lavandulyl isobutyrate, epoxy-α-terpenyl acetate, hexenyl isobutanoate, hexyl acetate, hexyl isobutyrate were detected. These traces compounds, particularly bornyl acetate, and lavandulyl acetate, previously reported in *Lavandula* species, contribute to the characteristic sweet, floral and fruity aroma of lavender oil^36^.

### Fatty acid/ester

Fatty acids and fatty acid esters (24.4%) were detected at a relative amount in L-Imt at (Table 1), while remaining negligible in the other products. Such elevated levels are not characteristic for profiles of authentic lavender essential oil and may reflect the presence of added carrier oils, formulation additives, solvent extraction residues. The presence of compounds such as isopropyl linoleate (Peak 50) and oleyl acetate (Peak 54) suggests the introduction of non-volatile lipidic materials that are commonly associated with vegetable carrier oils or cosmetic formulation ingredients^47^. Isopropyl linoleate is an ester derived from linoleic acid and is widely used in cosmetic formulations as an emollient^48^. Similarly, oleyl acetate originates from oleyl alcohol and is typically found in lipid-based ingredients rather than in distilled essential oils. The detection of these compounds at relatively high proportions in L-Imt therefore cannot be explained by natural biosynthesis in lavender plants. Instead, their presence strongly suggests commercial dilution of the essential oil with carrier oils or lipid-based additives, a practice sometimes used to reduce production costs or modify product viscosity. Consequently, while minor compositional differences between lavender oils may arise from natural plant variability, the substantial abundance of fatty acid derivatives observed in L-Imt is more consistent with intentional dilution or adulteration rather than natural variation.

### Aliphatic hydrocarbons

Aliphatic hydrocarbons were detected in L-Imt (7.09%) and L-Rag (29.32%). Pentatriacontane (Peak 42) was identified in both samples L-Imt (3.8%) and L-Rag (2.11%). It demonstrated antioxidant and anticancer effects against different cancer cell lines^49^. Whereas tetratetracontane (Peak 48) was only detected in L-Rag at a relative abundance (5.65%). It is of medicinal significance for its anti-inflammatory, antibacterial, and antiulcer activities^50^. The presence of these aliphatic hydrocarbons indicates the presence of impurities, wax residues, or wax residues during the processing of the volatile oil, which influences the compositions of the lavender volatile profile.

### Terpenes

Monoterpene compounds were detected at low levels (1.14–2.45%) in all samples except for L-Sha, with L-Rag sample at the highest proportion. The enantiomers α- and β-pinene (Peaks 1, and 4) are identified only in L-Imt sample. Previous studies have indicated that these enantiomers possess antimicrobial activity against several pathogens, showing synergistic effect when combined with commercial antibiotics, enhancing efficacy and decreasing toxicity^51^. 1,8-cineole (Peak 9), an oxide terpene, was detected in all products (3.44–5.79%) with the highest abundance in L-Nef. It is often incorporated into flavored products, cosmetics, and fragrances linked to its delightful aroma and taste^52^. Additional oxidative compounds as citronellol epoxide, Caryophyllene oxide, α-Limonene diepoxide, were observed at trace levels in L-Imt only, suggesting possible oxidative during the process of manufacturing or the storage^53^.

According to International Organization for Standardization (ISO 3515: 2002) for *Lavandula angustifolia* essential oil, acceptable range for linalool and linalyl acetate are 20–45% and 25–46% respectively^54^. In the present study, L-Sha and L-Rag close to the range of both key markers (linalool and linalyl acetate)^54^.

In contrast, L-Imt and L-Nef exhibited substantially reduced levels of key authenticity markers together with elevated amounts of non-characteristic constituents. While some compositional differences may arise from natural factors such as plant origin, chemotype variation, or processing conditions, the occurrence of compounds such as fatty acid esters and synthetic glycols cannot be explained solely by natural plant metabolism. These compounds are more plausibly associated with commercial dilution, formulation additives, or adulteration practices. Therefore, the observed deviations in these samples likely reflect quality compromise or modification of the original essential oil rather than simple botanical variability and underscore the importance of routine GC–MS profiling for authenticity assessment and quality control.

## Multivariate data analysis

The differences between lavender oil samples based on their volatile chemical composition were assessed by using multivariate data analysis tools.

### Unsupervised PCA analysis

The volatiles differences between four commercially available lavender oil samples were evaluated using multivariate data analysis using principal component analysis (PCA) and hierarchical clustering analysis (HCA) (Fig. 3).

The HCA dendrogram separating the samples into two distinct clusters (Fig. 3A), with Nef samples clustered separately in group 1, while the remaining oils samples grouped together at group 2. Similarly, the PCA score plot (Fig. 3B) of lavender oil volatiles showed discrimination of Nef clusters at the left side of PC1, in contrast to the other three samples that clustered towards the right side of PC1. The corresponding loading plot (Fig. 3C) revealed that 2,4 pentanediol and dipropylene glycol were more enriched in Nef samples versus abundance of linalool, camphor, and linalyl acetate in Sha samples helped in its segregation at lower right side, in addition to tetratriacontane and eicosane accounted for segregation of Rag samples at upper right side.

### Supervised OPLS-DA analysis

**Fig. 4:**
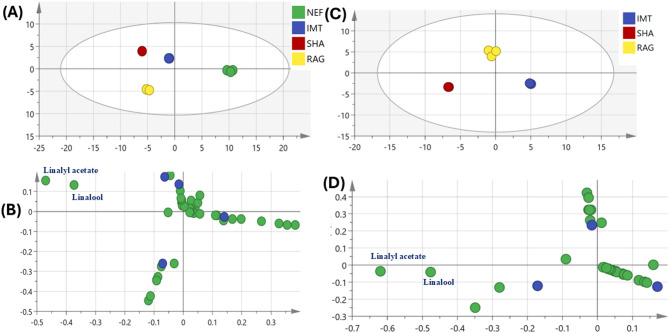
(**A**) OPLS-DA score plot based on GC–MS volatile metabolite profiles of four lavender samples (n = 3). (**B**) Corresponding loading plot showing variables contributing to group discrimination in the OPLS-DA model. (**C**) OPLS-DA score plot of three lavender samples (n = 3) following exclusion of Nef samples. (**D**) Corresponding loading plot.

Volatile profiles of lavender oil samples using supervised OPLS-DA model (Fig. 4) were further employed to assess their variation by constructing a model to assess relationship between different sources. OPLS-DA score plot (Fig. 4A) revealed segregation of Nef sample at the right side and the other samples at the left side.

**Fig. 5:**
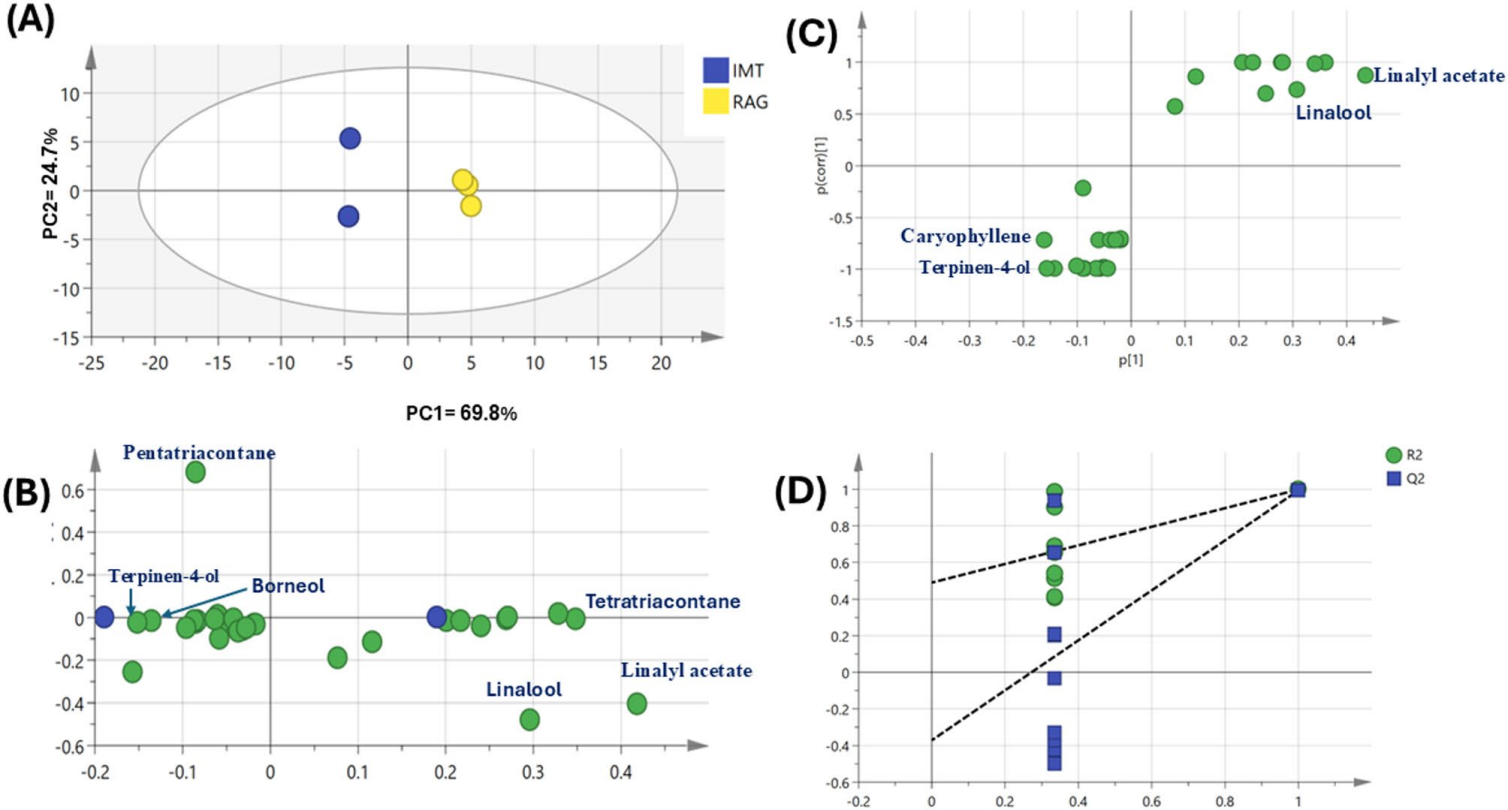
(**A**) OPLS-DA score plot derived from modeling volatile metabolites of Imt versus Rag lavender samples (n = 3). (**B**) The respective loading plot shows the discriminating component of the OPLS-DA model. (**C**) The respective loading S-plots of the discriminating component of the OPLS-DA model. (**D**) Permutation plot (n = 20) R2 (0.0, 0.454) and Q2 (0.0, − 0.48).

The corresponding loading plot (Fig. 4B) indicated an enrichment of 2,4 pentanediol and dipropylene glycol in Nef samples which contributed to their segregation on the right side. Other OPLS-DA model was employed for three samples (Imt, Rag, and Sha) after exclusion of Nef samples (Fig. 4C) showed Q^2^ = 0.84 and 0.99 and R^2^ = 0.97 and 0.99 and P value less than 0.04, indicating the model predictability and showed better discrimination between the samples. According to the loading plot (Fig. 4D), it was observed that linalyl acetate and linalool are enriched in Sha samples and helped in its discrimination from other samples. OPLS-DA model was employed for Imt samples versus Rag samples (Fig. 5A) showed better discrimination between the two samples with Imt samples were clustered at the left side and Rag samples at the right side. The respective loading plot (Fig. 5B) revealed that linalyl acetate, linalool, and tetratriacontane are enriched in Rag samples and helped in its discrimination, while Terpinen-4-ol, Caryophyllene, Borneol, Pentatriacontane were enriched in Imt samples. The respective loading S-plot (Fig. 5C) revealed esters and alcohols specially linalyl acetate and linalool are enriched in Rag samples and helped in its discrimination, while alcohols and sesquiterpenes including terpinen-4-ol and caryophyllene were enriched in Imt samples. The model permutation was shown in Fig. 5D.

**Fig. 6:**
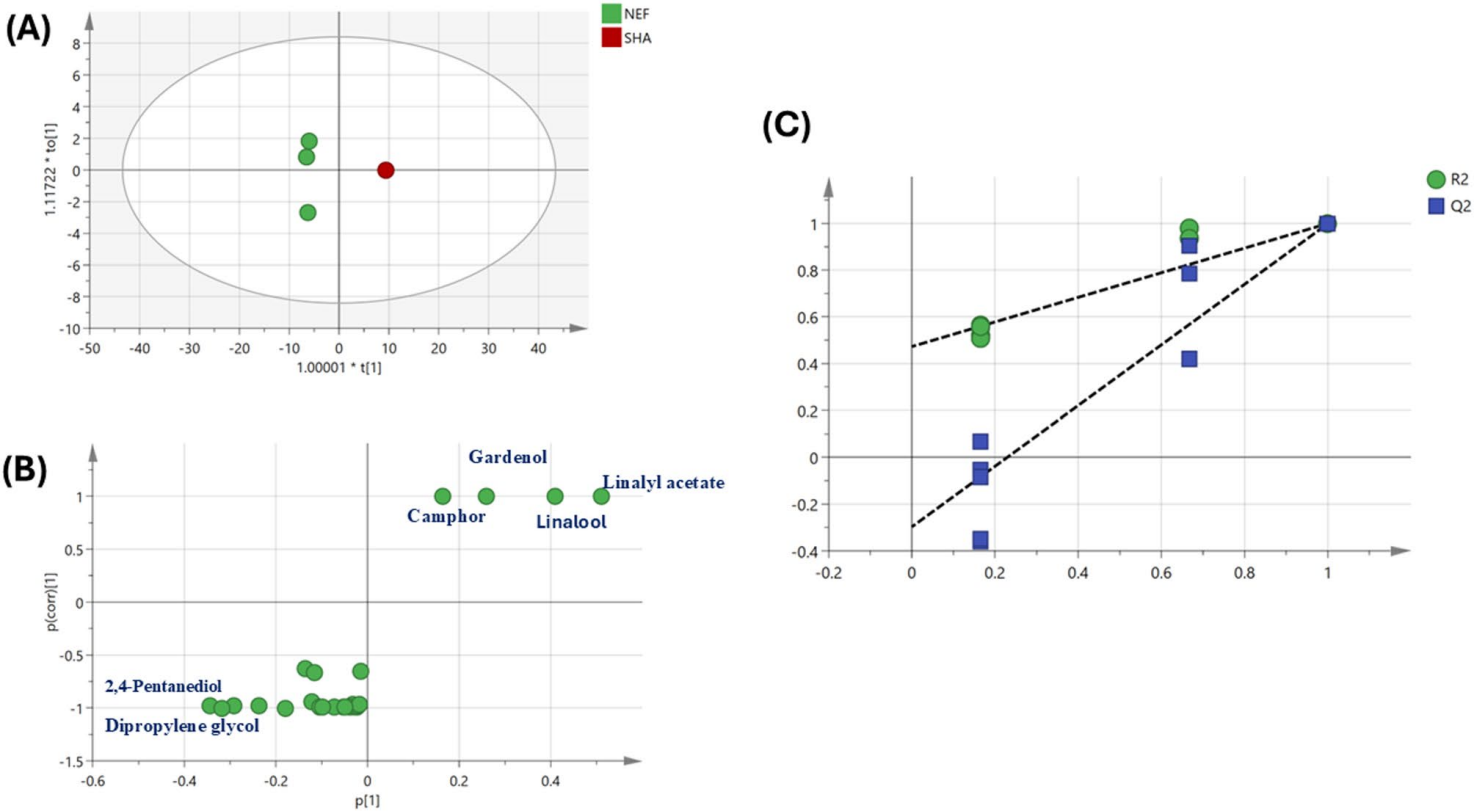
(**A**) OPLS-DA score plot derived from modeling volatile metabolites of Nef versus Sha lavender samples (n = 3). (**B**) The respective loading plot shows the discriminating component of the OPLS-DA model. (**C**) Permutation plot (n = 20) R2 (0.0, 0.472) and Q2 (0.0, − 0.298).

Another OPLS-DA model was employed for Nef samples versus Sha samples (Fig. 6A) showed better discrimination between the two samples with Nef samples were clustered at the left side and Sha samples at the right side. The model showed Q^2^ = 0.98 and 0.99 and R^2^ = 0.99 and 0.99 and *P* value less than 0.05, indicating the model predictability and showed better discrimination between the samples The respective loading S-plot (Fig. 6B) revealed that linalyl acetate, linalool, gardenol and camphor were enriched in Sha samples and helped in its discrimination, while 2,4 pentanediol and dipropylene glycol were more enriched in Nef samples. The model permutation was shown in Fig. 6C.

## Comparison between samples according to extraction method

The analyzed oils were reportedly obtained using two industrial extraction approaches: steam distillation (L-Sha and L-Rag) and solvent extraction (L-Imt and L-Nef). While chromatographic peak intensities appeared higher in some solvent-derived samples, these differences reflect detector response rather than absolute abundance. Therefore, Table 1 reports normalized relative percentages calculated from integrated peak areas of the total ion chromatogram (TIC). Multivariate analysis using the OPLS-DA model based on GC–MS volatile profiles revealed clear discrimination among the samples (Fig. 4A). In general, the solvent-extracted products (L-Imt and L-Nef) clustered separately from the steam-distilled oils (L-Sha and L-Rag), indicating differences in their overall volatile composition. Such variations may arise from differences in extraction techniques, since steam distillation typically enriches volatile monoterpenes and their oxygenated derivatives, whereas solvent-based extraction methods may co-extract less volatile or lipid-derived constituents. However, the compositional deviations observed in certain samples cannot be attributed solely to extraction technique. For example, the detection of substantial amounts of fatty acid esters in L-Imt and synthetic glycols such as 2,4-pentanediol in L-Nef is atypical for authentic lavender essential oil and more consistent with the presence of carrier oils or formulation additives. When the L-Nef sample was excluded from the model (Fig. 4C and D), discrimination among the remaining three oils was further improved, suggesting that this sample exhibited a markedly distinct chemical profile. These findings indicate that while extraction methods may contribute to some variability in volatile composition, the presence of non-characteristic compounds in certain samples reflects commercial modification or dilution rather than natural extraction-related variation alone. This observation further highlights the value of GC–MS profiling combined with chemometric analysis for distinguishing authentic essential oil profiles from potentially adulterated commercial products.

## Conclusion

Lavender essential oil is widely valued for its aromatic and functional properties; however, its commercial quality is strongly influenced by botanical origin, cultivation conditions, processing methods, and potential post-production modifications. The present study revealed substantial compositional variability among four commercially available lavender oils in the Egyptian market. Notably, L-Sha and L-Rag exhibited relatively higher levels of the key authenticity markers linalool and linalyl acetate, showing closer conformity to recognized *Lavandula angustifolia* compositional standards. In contrast, L-Imt and L-Nef displayed markedly different chemical profiles characterized by reduced levels of these characteristic monoterpenes together with the presence of non-typical constituents such as fatty acid esters and synthetic glycols, suggesting possible dilution or formulation with carrier oils or additives. These findings demonstrate that while some variations may arise from natural factors or processing conditions, the occurrence of non-characteristic compounds can indicate compromised product authenticity. The lack of manufacturer-disclosed information regarding plant origin, cultivation conditions, and industrial processing represents a limitation when interpreting compositional differences among commercial oils, as these factors are known to significantly influence essential oil chemical profiles. Moreover, a limitation of this study is that the analyzed products were produced using different industrial extraction, which may contribute to part of the observed compositional variability. Overall, the results highlight the value of GC–MS profiling combined with chemometric analysis as a robust approach for authenticity verification, detection of potential adulteration, and quality control of commercial lavender oils. Such analytical strategies can support regulatory monitoring and help ensure product consistency and consumer confidence in the essential oil market. While GC–MS profiling provides detailed compositional information, future studies should also incorporate physicochemical parameters specified in regulatory standards, such as refractive index and density, to achieve a more comprehensive quality evaluation of lavender oil.

## Material and methods

### Samples collection

Four commercial lavender essential oils (*Lavandula angustifolia* Mill.) were purchased from the local market in Cairo, Egypt, in September 2024. The products were produced and marketed by four different companies: Imtenan, Nefertari, Shana, and Ragab. The selected brands represent widely available commercial products in the Egyptian markets, making them relevant for consumer-level quality assessment. All samples were stored in dark amber containers at 23–25 °C and 30–40% relative humidity until analysis. The samples were coded as follows: L-Imt (Imtenan), L-Nef (Nefertari), L-Sha (Shana), and L-Rag (Ragab). According to the product labels, L-Sha and L-Rag were obtained via steam distillation, whereas L-Imt and L-Nef were extracted using solvent extraction methods. It should also be noted that detailed information regarding the geographical origin of the plant material, cultivation conditions, and industrial extraction parameters was not disclosed by the manufacturers. For each commercial oil, three independent samples were analyzed under identical conditions to serve as biological replicates, ensuring reproducibility of the results.

### Sample preparations and GC–MS analysis

Each lavender essential oil sample (5 μL) was diluted to 1 mL with analytical-grade *n*-hexane (0.5% v/v). The solutions were thoroughly vortex-mixed to ensure homogeneity. For GC–MS analysis, 1 μL of each diluted sample was directly injected in split mode with a split ratio of 1:15. All samples were prepared and analyzed under identical conditions to maintain consistency and reproducibility, allowing reliable comparative profiling of the volatile components. The use of direct liquid injection ensures that both volatile and semi-volatile constituents of the oils are captured for analysis.

GC–MS analyses were performed at the Pharmacognosy Department, Faculty of Pharmacy, Ain Shams University, Cairo, Egypt, using a Shimadzu GCMS-QP2010 system (Kyoto, Japan) fitted with an Rtx-5MS fused-bonded column (30 m × 0.25 mm i.d., 0.25 μm film thickness; Restek, USA) and a split/splitless injector. The oven temperature program was initially set 50 °C for 3 min (isothermal), followed by a ramp of 5 °C/min to 300 °C, which was maintained for 10 min (isothermal). The injector was set at 280 °C. Helium was used as the carrier gas at a flow rate of 1.37 ml/min. Mass spectra was recorded under electron ionization (EI) mode at 70 eV, with a filament filament emission current of 60 mA and an ion source temperature, 220 °C.

### Metabolites identification and relative quantification

Volatile compounds were identified by comparing their retention indices (RI) and retention times (RT) relative to series of n-alkanes (C6–C20), together with mass spectral matching with the NIST mass spectral libraries^55^. Prior to library matching, chromatographic peaks were deconvoluted using the Automated Mass Spectral Deconvolution and Identification System (AMDIS; www.amdis.net). Compound identification was accepted based on spectral similarity with the NIST library using a minimum match factor threshold together with consistency between experimental and literature retention indices^55^. The relative abundance of each compound was calculated using peak area normalization of the total ion chromatogram (TIC) and expressed as relative percentage composition. It should be noted that these values represent semi-quantitative relative abundances rather than absolute concentrations, as no internal standards or detector response correction factors were applied. Nevertheless, this approach is widely used in essential oil profiling and provides reliable comparative information on the distribution of volatile constituents among samples analyzed under identical analytical conditions. Compounds were listed in Table 1 in order of their elution and their percentages were obtained by peak-area normalization^56^.

#### Multivariate data analysis

The data were analyzed using principal component analysis (PCA), hierarchical clustering analysis (HCA), and orthogonal partial least squares discriminant analysis (OPLS-DA) with SIMCA-P software version 13.0 (Umetrics, Umeå, Sweden). Potential markers were identified through examination of the loading plots. Prior to analysis, all variables were mean-centered and scaled according to Pareto variance. Model validity was assessed based on diagnostic indices, including Q^2^ and R^2^ values, *p*-values, and permutation testing.

#### Statistical analysis

All findings are shown as the mean with corresponding standard deviation (Sd).

## Supplementary Information

Below is the link to the electronic supplementary material.

Supplementary Material 1

## Data Availability

All data generated or analyzed during this study are included in this published article.
