# Supplementary material for: Uncovering adulteration and quality variations in commercial lavender essential oils from the Egyptian market using GC–MS and chemometrics
Source: Sci Rep. 2026 Apr 18;16:12735. doi: 10.1038/s41598-026-45972-6 (PMC13091794; doi:10.1038/s41598-026-45972-6)
Supplement: Supplementary file 1 — Supplementary Material 1 [file 41598_2026_45972_MOESM1_ESM.docx]

**Uncovering adulteration and quality variations in commercial lavender essential oils from the Egyptian market using GC-MS and chemometrics**

**Mostafa B. Abouelela^1^, Eman M. El-Taher^1^, Enas M. Shawky^1^, Mostafa H. Baky^1^***

*^1^* *Pharmacognosy Department, College of Pharmacy, Egyptian Russian University, Badr City 11829, Cairo, Egypt.*

*Corresponding author:

**Mostafa H. Baky**

*Pharmacognosy Department, College of Pharmacy, Egyptian Russian University, Badr City 11829, Cairo, Egypt.*
E-mail addresses: [dr_mostafa1984@yahoo.com](mailto:dr_mostafa1984@yahoo.com), [mostafa-hasan@eru.edu.eg](mailto:mostafa-hasan@eru.edu.eg)

Tel: +201007906443


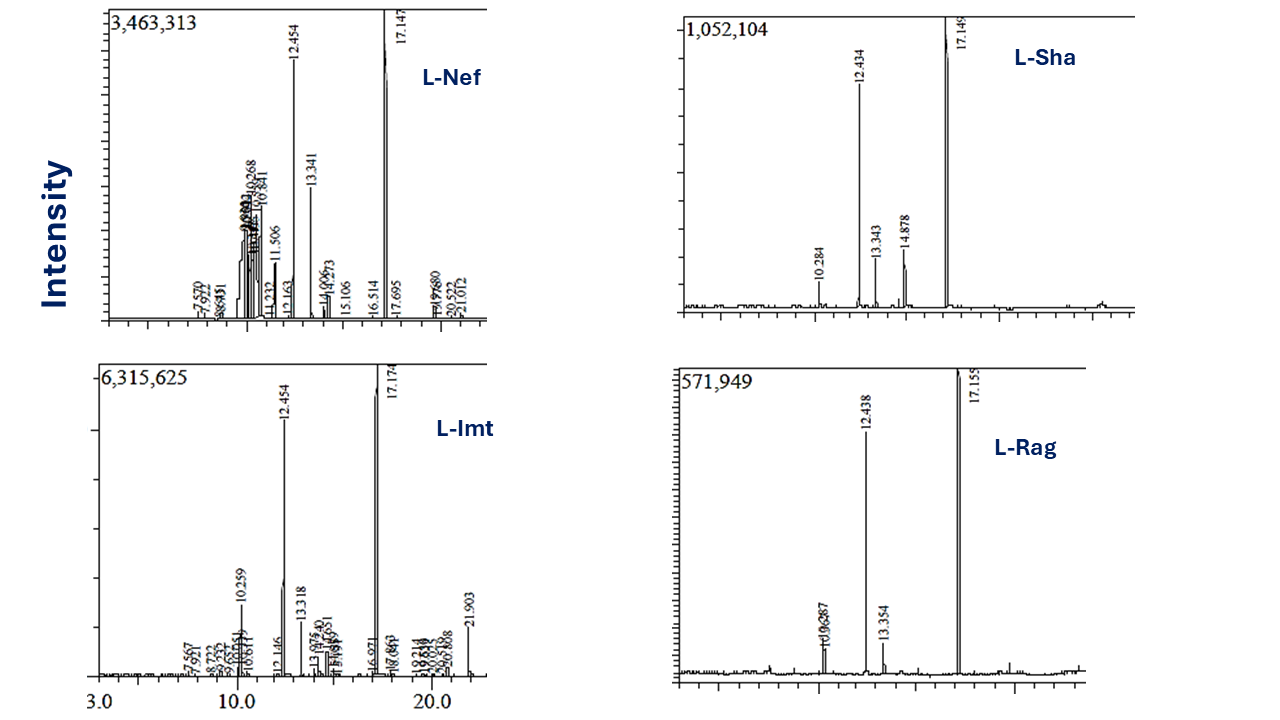


**Figure S1.** Enlarged GC-MS chromatograms (3-20 min) of the four commercial lavender oil samples showing the separation of the major volatile constituents.
